# Antibodies as clinical tools for tuberculosis

**DOI:** 10.3389/fimmu.2023.1278947

**Published:** 2023-12-14

**Authors:** Sophie McIntyre, Jeffrey Warner, Catherine Rush, Hillary A. Vanderven

**Affiliations:** ^1^ Biomedical Sciences and Molecular Biology, College of Public Health, Medical and Veterinary Sciences, James Cook University, Douglas, QLD, Australia; ^2^ Australian Institute of Tropical Health and Medicine, James Cook University, Douglas, QLD, Australia; ^3^ Department of Microbiology and Immunology, Peter Doherty Institute for Infection and Immunity, University of Melbourne, Parkville, VIC, Australia

**Keywords:** tuberculosis, antibodies, immunotherapy, vaccines, mycobacteria, serodiagnostics, humoral immunity, monoclonal antibodies

## Abstract

Tuberculosis (TB) is a leading cause of morbidity and mortality worldwide. Global research efforts to improve TB control are hindered by insufficient understanding of the role that antibodies play in protective immunity and pathogenesis. This impacts knowledge of rational and optimal vaccine design, appropriate diagnostic biomarkers, and development of therapeutics. Traditional approaches for the prevention and diagnosis of TB may be less efficacious in high prevalence, remote, and resource-poor settings. An improved understanding of the immune response to the causative agent of TB, *Mycobacterium tuberculosis* (*Mtb*), will be crucial for developing better vaccines, therapeutics, and diagnostics. While memory CD4+ T cells and cells and cytokine interferon gamma (IFN-g) have been the main identified correlates of protection in TB, mounting evidence suggests that other types of immunity may also have important roles. TB serology has identified antibodies and functional characteristics that may help diagnose *Mtb* infection and distinguish between different TB disease states. To date, no serological tests meet the World Health Organization (WHO) requirements for TB diagnosis, but multiplex assays show promise for improving the sensitivity and specificity of TB serodiagnosis. Monoclonal antibody (mAb) therapies and serum passive infusion studies in murine models of TB have also demonstrated some protective outcomes. However, animal models that better reflect the human immune response to *Mtb* are necessary to fully assess the clinical utility of antibody-based TB prophylactics and therapeutics. Candidate TB vaccines are not designed to elicit an *Mtb*-specific antibody response, but evidence suggests BCG and novel TB vaccines may induce protective *Mtb* antibodies. The potential of the humoral immune response in TB monitoring and control is being investigated and these studies provide important insight into the functional role of antibody-mediated immunity against TB. In this review, we describe the current state of development of antibody-based clinical tools for TB, with a focus on diagnostic, therapeutic, and vaccine-based applications.

## 1 Introduction

Tuberculosis (TB) remains a global health crisis, primarily affecting low- and middle-income countries (LMICs). With the rise of COVID-19 and the associated disruptions to healthcare services in LMICs, the TB crisis has only worsened (1). WHO estimates that 10.6 million people were infected with *Mtb* and 1.6 million died of TB in 2021 (2). Given the continued and significant global health burden of TB, the need for improved diagnosis, prevention, and treatment of *Mtb* infection is increasingly urgent, particularly in resource poor settings.

While a spectrum of TB presentations exists, it typically presents as a pulmonary disease (3). People with active pulmonary TB (PTB) produce airborne respiratory droplets through coughing and sneezing that contain *Mtb* bacteria. Inhalation of these infectious droplets can lead to *Mtb* infection and TB disease. Infectious droplets travel to the lower respiratory tract where *Mtb* encounters innate immune cells including alveolar macrophages (AMs), neutrophils, dendritic cells (DCs), and monocytes (4). The AMs phagocytose *Mtb* in the lower airways and alveolar spaces, allowing *Mtb* to preferentially infect and replicate within them. Uptake by AMs provides *Mtb* with an intracellular niche to grow and replicate that is largely protected from extracellular immune mediators (like antibody and complement) (4). However, active TB (ATB) is not always limited to the lungs and *Mtb* can disseminate to nearly any organ system including the lymph nodes, pleurae, gastrointestinal tract, skeleton, central nervous system, and the genitourinary tract (5, 6). This is termed extrapulmonary TB (EPTB), which accounts for ~15% of all *Mtb* infections (6). Although it can occur in immunocompetent individuals, there is a much higher incidence of EPTB in individuals with comorbidities like human immunodeficiency virus (HIV) infection (7). The pathogenesis of EPTB involves migration of *Mtb* into lymph nodes and eventually through the bloodstream to distal organs or tissues (8). As such, EPTB may present with no evidence of pulmonary *Mtb* infection (9).

**Figure 1:**
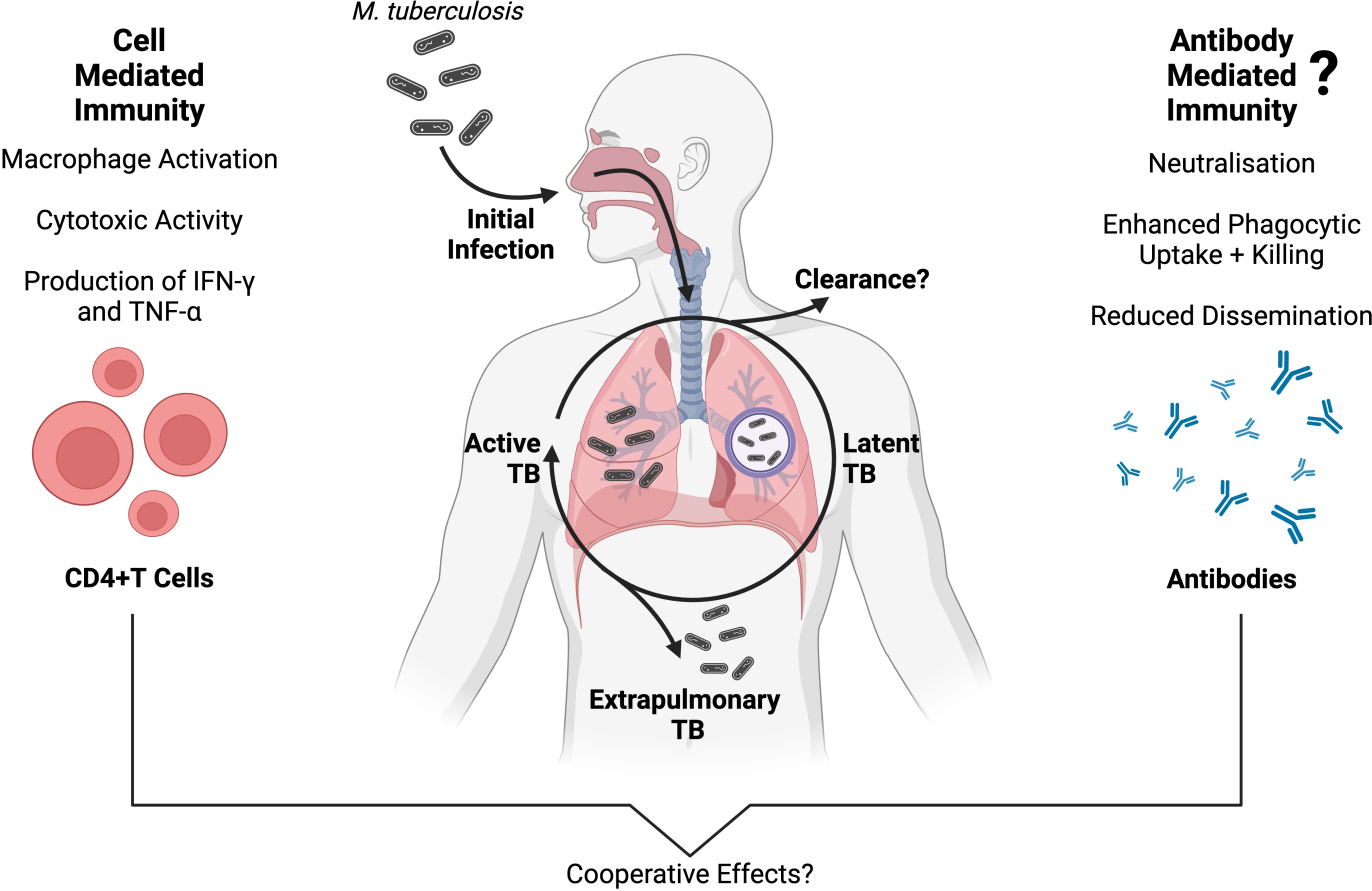
An overview of adaptive immunity to *Mtb* infection, and the progression of TB disease presentations. Protective cell-mediated immunity against *Mtb* is well established, and primarily involves CD4+ T cells producing inflammatory cytokines (such as IFN-γ and TNF-α) which activate the antimicrobial functions of *Mtb*-infected macrophages (10, 11). Conversely, antibody-mediated immunity has a less clear role in *Mtb* infection, although a growing body of evidence supports a protective association between *Mtb*-specific antibodies and TB disease outcomes (12–16). While the two branches of adaptive immunity have long been studied separately in TB, they are interconnected (17) and multiple independent studies suggest that cell- and antibody-mediated immunity are working cooperatively to protect against *Mtb* infection, inhibit *Mtb* growth and reduce disseminated TB disease (14, 18–20). Created with BioRender.com.

The two primary outcomes that result from *Mtb* infection are early clearance of *Mtb* by the innate immune system, or latent persistence within lung granulomas (**Figure 1**). Innate immune clearance of *Mtb* occurs in a proportion of the human population, possibly due to a strong pro-inflammatory cytokine response and decreased recruitment of monocytes (21, 22). Individuals who cannot clear *Mtb* will go on to develop a latent TB infection (LTBI) (3). In LTBI, an adaptive immune response develops. The adaptive immune response to *Mtb* is characterised by T cell priming and the presence of *Mtb*-specific CD4+ T cells that secrete the macrophage-activating cytokine interferon gamma IFN-γ (**Figure 1**) (10). T cell priming occurs when infected DCs and macrophages present processed *Mtb* antigens to T cells in the lymph nodes (10). Both B and T lymphocytes, as well as innate immune cells, contribute to the formation of lung granulomas. These contain *Mtb* in a non-replicating latent phase for months to years (23). Development of ATB is estimated to occur in 5-15% of individuals infected with LTBI in the first few years (24).

The interplay between *Mtb* and the host adaptive immune response is complex and may help determine the course of *Mtb* infection as well as the type of TB disease presentation (25, 26). It is becoming increasingly evident that a complete understanding of the immune response to *Mtb* requires the examination of both cell-mediated and humoral immunity (**Figure 1**) (17, 27, 28). In the last few decades, our understanding of TB immunity has advanced significantly, but the role of antibodies during *Mtb* infection is not yet fully characterised. Human infection with *Mtb* is known to induce a robust humoral immune response and the production of *Mtb*-specific antibodies against a variety of antigens (29). Overall, *Mtb*-specific antibody titres appear to increase with TB disease burden, likely because of increased antigen availability (12, 30–32). There is a growing interest in the antibody response to *Mtb* and there is increasing evidence that different TB disease states are associated with distinct antibody specificities and functions (12).

**Figure 2:**
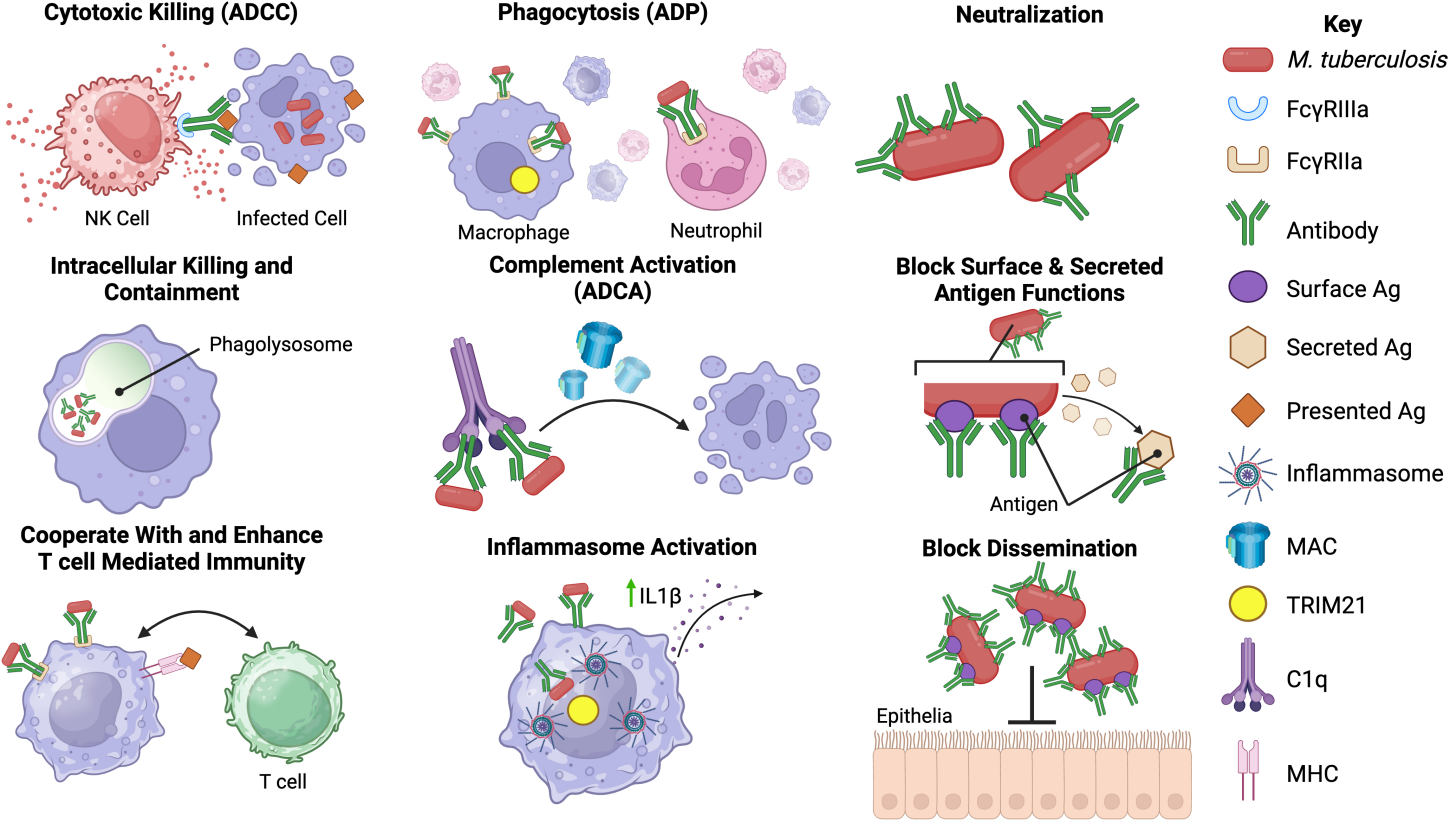
Potential functions of antibodies against *Mtb*. Antibodies may have an array of functions in the immune response against *Mtb* infection. The most well-known of these is neutralization of extracellular *Mtb* and secreted products via the antibody fragment antigen-binding (Fab) domain. Binding of antibody to *Mtb* may block entry of *Mtb* into host cells, aid in intracellular killing or control of replication, enhance phagocytosis by opsonizing *Mtb*, prevent the actions of secreted proteins and help prevent dissemination from the lungs (35–38). The fragment crystallizable (Fc) region of *Mtb* antibodies may also play a role in the immune response to *Mtb*. Fc-mediated antibody functions including antibody-dependent phagocytosis (ADP), antibody-dependent complement activation (ADCA), antibody-dependent cellular cytotoxicity (ADCC) and inflammasome activation may occur during *Mtb* infection (12, 13, 39, 40). Possible interactions with cell-mediated immunity, including enhancement of T-cell responses in the presence of specific antibodies, have been reported (14, 41). However, the precise mechanisms underlying some of the proposed *Mtb* antibody functions are not known or poorly defined in *Mtb* infection. Created with BioRender.com.

Antibody-mediated immunity (AMI) against *Mtb* occurs despite its largely intracellular lifecycle, which partially shields mycobacteria from humoral mediators. There are, however, multiple instances during its progression where *Mtb* and its antigens are extracellular, including during the initial infection, within the necrotic tissue of granulomas, and during the death of *Mtb*-infected cells (33, 34). Antibodies can also form immune complexes with soluble antigen or bind to infected cells and mediate effector functions, such as phagocytosis and targeted killing of infected cells, through engagement of Fc receptors on immune cells (**Figure 2**) (42).

There is significant heterogeneity in the human antibody response to *Mtb*, and the proportion of *Mtb*-infected individuals that make antibodies against *Mtb* antigens varies widely between studies (13, 29, 43). As the antibody response to *Mtb* is so diverse, no consensus has been reached regarding the immunodominant antigens and target epitopes for humoral immunity. Consequently, studies measure responses to diverse panels of *Mtb* antigens and different isotypes/subclasses, making it challenging to compare results across studies (13). Associations between TB disease and antibody titre, isotype, or specificity are not well understood, but some progress has been made towards uncovering protective and pathological features of the antibody response to *Mtb* (44). Several immunodominant antigens targeted by *Mtb* antibodies have been identified including heat shock protein (HspX), lipoarabinomannan (LAM), arabinomannan (AM), heparin-binding hemagglutinin adhesin (HBHA), phosphate transporter subunit (PstS1), early secreted antigen (ESAT-6), and culture filtrate protein (CFP-10) (45, 46). These and many other target antigens play important roles in *Mtb* virulence, such as immunomodulation and adhesion to host cells (47–50). *Mtb*-specific antibodies could play a role in disrupting the functions of key virulence factors and may, therefore, influence TB disease progression.

**Figure 3:**
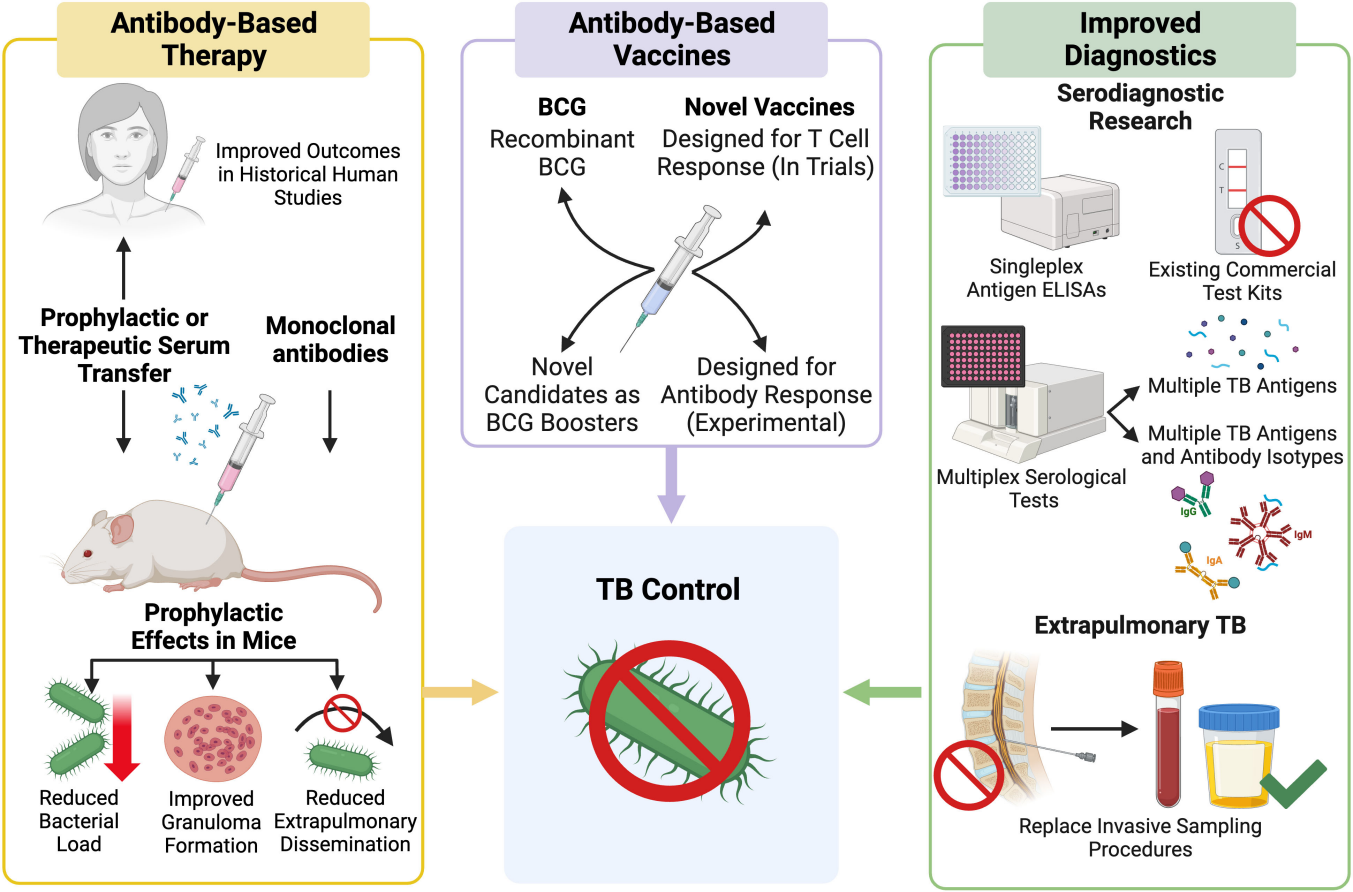
Outline of current research pathways and progress towards use of *Mtb* antibodies in a clinical setting. Created with BioRender.com.

Advancing our understanding of AMI during *Mtb* infection may provide new insights into the different TB disease states and may contribute to the development of clinical applications for *Mtb*-specific antibodies (**Figure 3**). This could include the discovery of novel antibody biomarkers to improve TB diagnosis or to aid in the identification individuals at risk of serious or progressive disease (51, 52). Antibodies with protective functions during *Mtb* infection could form the basis for new TB therapies and vaccines (53). Development of a more effective vaccine, shorter drug treatments, and more accessible diagnostics are all considered vital to the WHO End TB STRATEGY (52, 54). This review examines the clinical applications of *Mtb* antibodies including serodiagnostics, antibody-based therapeutics, and the humoral response to TB vaccines (**Figure 3**). Herein, we describe the state of development of serological tools to control and treat TB as well as the limitations that exist to their implementation, especially in TB endemic regions.

## 2 *Mtb* antibodies as biomarkers in TB diagnosis

### 2.1 Existing TB diagnostics

Current methods for diagnosing TB in LMICs are limited in their sensitivity and cannot reliably distinguish different forms of TB (55). Given the high prevalence of TB, particularly LTBI and EPTB, in LMICs there is a need for rapid, sensitive, and accessible diagnostic tools. The ideal TB diagnostic test would be able to identify TB cases and differentiate between TB disease states (56, 57). Serological tests for *Mtb* may be advantageous due to their low cost and ease of use (58). Rapid serologic tests for COVID-19 have demonstrated the potential of antibody-based diagnostics for transmission control, although many COVID-19 tests were antigen based (59).

Reliance on acid fast bacilli staining (AFB) of sputum for TB diagnosis is common in LMICs due to relative affordability, rapid turnaround, and accessibility (58). Variable sensitivity (ranging from 20-80%) is a major drawback of AFB testing, which routinely misses *Mtb-*infected individuals with low or no lung bacillary load who may have subclinical PTB, LTBI, or EPTB (60, 61). The inability to reliably detect these *Mtb*-infected individuals contributes to higher TB transmission and mortality rates. Serological tests may circumvent these limitations by detecting *Mtb*-specific antibodies that correspond to different TB disease states. Alternative methods of diagnosing TB exist, including PCR-based Xpert *MTB*/RIF and bacterial culture. However, both PCR- and culture-based TB diagnostics have notable drawbacks (58). Culture-based diagnosis of *Mtb* infection is considered the gold-standard by the WHO, but bacterial culture has a long turnaround time and laboratory infrastructure requirements that limit its use in LMICs (62). The Xpert *MTB*/RIF assay is also WHO approved and used for fast molecular detection of *Mtb*. This PCR-based test has a similar specificity and sensitivity to *Mtb* culture, but its use in LMICs is limited by high cost and infrastructure requirements such as access to electricity (63–65).

The optimal biomarker test for TB would be simple, portable, rapid, and applicable to a broad range of *Mtb*-infected individuals, including those who are AFB negative (28). The WHO emphasises that new diagnostic tests for TB should have specificity and sensitivity similar to (or greater than) that of the Xpert *MTB*/RIF PCR assay for adults with ATB (both PTB and EPTB) and be able to distinguish between ATB, past TB and LTBI (28, 66). The only WHO-approved non-sputum-based point of care (POC) TB test measures *Mtb* LAM antigen in urine using a lateral-flow assay (LF-LAM). However, variable sensitivity (17.8-80.3%) and specificity (87.7-99%) have been reported for the LF-LAM assay (67–69), and it is only recommended for individuals with low CD4+ T cell counts (e.g. HIV infected) due to low sensitivity (< 20%) in immunocompetent individuals (70, 71).

### 2.2 *Mtb* antibodies as diagnostic biomarkers of ATB and LTBI

To date, the use of TB serodiagnostic tests has been contentious due to the poorer sensitivity and specificity of commercially available kits compared to conventional testing methods (72). As a result, the WHO has recommended against the use of current commercial serological tests for *Mtb* diagnosis (73–75). Despite this, the WHO has encouraged ongoing research efforts to develop new antibody-based TB diagnostics due to the advantages of serological testing formats in LMICs.

Current single antigen *Mtb* serodiagnostic tests have demonstrated low sensitivity and their development may be complicated by the heterogenous nature of the antibody response to human *Mtb* infection (46, 73, 76, 77). These diagnostic tests detect antibodies against various *Mtb* antigens including Mtb mammalian cell entry protein 1A (Mce1A), PstS1, proline-proline-glutamic acid protein 17 (PPE17), and A60 (**Table 1**). The detection of antibodies against multiple *Mtb* antigens and/or of multiple antibody isotypes may be more effective, and this approach may improve sensitivity (53). Multiple studies have aimed to identify which combinations of *Mtb* antibodies produce the optimal sensitivity and specificity for identifying TB across a broad range of *Mtb*-infected individuals. Serological assays would be particularly useful for TB groups that are more challenging to diagnose including smear (or *MTB*/RIF) negative patients with active PTB, individuals with LTBI, young children, and people with HIV co-infection. Various testing formats including ELISA, lateral flow and multiplex microbead assays have been utilised, although no study has directly compared different assay formats thus far.

**Table 1:** Comparison of different serodiagnostic test formats and approaches for diagnosing *Mtb* from the last two decades.

Type of test	Ab Isotype and Specificities	Sensitivity	Specificity	Reference
Single Specificity and Isotype				
ELISA	IgG Mce1A	PTB 79.5%	84.4%	(78)
ELISA	IgG PstS1	PTB Smear Pos 29-82%	96%	(79)
ELISA	IgG PPE17	LTBI 65-86% ATB 69-94%	100%	(80)
ELISA	IgG A60	PTB 94% EPTB 84%	92%	(81)
Multiple Specificities				
Multiplex Microbead Assay	Rv3881c, PstS1, Rv0054, Rv3804c, HspX, Ag85b, Rv0129c, Rv1860, Rv1980c, Rv3874, Rv0831c, Rv2875, Rv3841, Rv1926c, ESAT6, Rv2878c	PTB Smear Pos 92-93% PTB Smear Neg 88%	79%	(82)
Multiplex Microbead Assay	ESAT-6, CFP-10, HspX, MPT53, MPT63	–	–	(83)
Multiplex Microbead Assay	IgG: Ag85B, Ag85A, Ag85C, PstS1-P38, Rv3881, BfrB, Rv3873 and Rv2878c	ATB 90.6%	88.6%	(84)
ELISA	IgG: Rv3881, PstS1, HspX,Ag85b, Rv1860, Rv3874, Rv2875, Rv3841, Rv1926c, MEMH37Rv and Rv1984	PTB Smear Pos 95% PTB Smear Neg 88%	91%	(85)
ELISA	IgG: Rv3871, Rv3876, and Rv3879	PTB 79.53%,	90.53%	(86)
ELISA	IgG: CFP-10, CFP-21, ESAT-6, MPT-64	PTB Smear Pos 52% PTB Smear Neg 43%	97%	(87)
ELISA	Rv0310c-E and Rv3425	PTB Sputum Pos 87.30%	73.68%	(88)
	Rv1255c-E and Rv3425	PTB Sputum Neg 87.30%	73.68%	
ELISA	IgG 88-kDa protein, Ag85C, MPT32	ATB Smear Pos 81% ATB Smear Neg 50%	98%	(89)
Protein Chip Array	IgG: LAM, 38-kDa, 16-kDa	ATB 93.1%	77.3%	(90)
Luciferase Immunoprecipitation	PstS1, Rv0831c, FbpA, EspB, bfrB, HspX and ssb	PTB 74%	96%	(91)
Lateral Flow Test	ESAT-6, CFP-10, *Mtb*8, *Mtb*48, MPB70, MPB83, HspX, PstS1, CFP10/ESAT-6 fusion, Acr1/MPB83 fusion	NHP PTB 90%	99%	(92)
Multiple Isotypes and/or Specificities				
ELISA	Anti-Tpx+L16 IgG, anti-Tpx IgG and anti-MPT64 IgA	ATB 95.2%	97.6%	(93).
ELISA	anti-LAM IgA, anti-LAM IgG, anti-Tpx IgG, anti-HSP16.3 IgG, anti-HSP20 IgA	ATB 81%	94%	(94)
	anti-LAM IgA, anti-LAM IgG, anti-Tpx IgA, anti-Tpx IgG, anti-Apa IgM	LTBI 81%	91.5%	
ELISA	HspX IgG, IgA, IgM	ATB Smear Pos 83%	93%	(95)

A lateral-flow POC test that detects antibodies against multiple *Mtb* antigens, including ESAT-6, CFP-10, HspX, and PstS (**Table 1**), in *Mtb*-infected non-human primates (NHPs) showed high sensitivity and specificity (90% and 99%) (92). This POC test also demonstrated no cross reactivity with infections from non-tuberculous (NTB) mycobacterium species. Immunodominant *Mtb* antigens, such as ESAT-6, CFP-10, and HspX, have also been tested in a multiplex format to detect *Mtb*-specific antibodies in NHPs with both ATB and LTBI (83). Antibodies against ESAT-6 and CFP-10 were consistently detected in NHPs with LTBI, while NHPs with ATB had detectable antibodies against ESAT-6 and CFP-10 as well as HspX. These findings suggest that HspX antibodies may help differentiate ATB from LTBI *in vivo*. This antibody-based multiplex assay also showed no cross reactivity with Bacille Calmette-Guérin (BCG) in vaccinated NHPs, which is highly desirable in TB endemic LMICs. Antibodies targeting ESAT-6, CFP-10 and HspX appear to be detectable in NHP with ATB and LTBI, but it is unclear whether they could reliably distinguish between TB disease states in humans (83). Further testing of serological POC and multiplex assays in human cohorts is required to fully assess their diagnostic potential. Two multiplex assays that detect a combination of different *Mtb*-specific human immunoglobulin Gs (IgGs) (**Table 1**) had high sensitivity and specificity for diagnosing both smear-positive and negative ATB cases (88-95% sensitivity and 88.6-91% specificity) (84, 85). This indicates that these serological tests could be useful for diagnosing TB irrespective of lung bacillary load, circumventing the issue of negative smear results in individuals with TB. Other antibody-based TB tests have reported well-defined and consistent antibody profiles in people with ATB but had much poorer sensitivity and specificity relative to those described above (**Table 1**).

Multiplex assays using cytokines and other inflammatory biomarkers can also be used to diagnose ATB (96). However, these assays may have relatively low specificity as other respiratory diseases can induce similar inflammatory responses. Since antibody-based biomarker assays often lack sensitivity, an assay combining these two biomarker types could be highly effective. To date, multiple groups have developed TB diagnostic tests including both cytokines and antibodies in an attempt to raise test specificity and sensitivity (97). A microarray chip assay for diagnosis of ATB in serum samples found that a combined biomarker assay had similar or improved sensitivity and specificity compared to conventional TB tests (98). The criteria used for ATB diagnosis were positive expression of inflammatory markers (I-309, IL-8 and MIG) or the presence of IgG antibody specific for at least one of the selected *Mtb* antigens (Ag14-16kDa, Ag32kDa, Ag38kDa, and Ag85B). Individuals with ATB were compared to healthy controls and people with other respiratory diseases. Overall, this combined test demonstrated high sensitivity, specificity, and accuracy (91.03%, 92.59% and 91.82%) for diagnosing people with ATB (98). A marked increase in sensitivity and accuracy was observed when using a combination of biomarker types compared to using either biomarker alone. Conventional tests, including interferon-gamma release assay (IGRA) and TB culture, were also performed for this population, but the combined biomarker test was superior for ATB diagnosis (98). More recently, another combined biomarker assay demonstrated improved performance over single biomarker assays (97). Discriminant analysis was performed with antibody ELISA data and multiplex cytokine and inflammatory marker profiles in serum and plasma. Individuals with culture confirmed ATB or suspected TB were compared to a control population with other respiratory diseases. Two different combinations of biomarker profiles were found to have high sensitivity and specificity for detecting ATB. The most promising biomarker combination (including MTP64 IgA, Tpx IgA + NCAM-1, vitronectin, CFH, ferritin and A2M) had a sensitivity of 95% and a specificity of 88.5% for ATB diagnosis (97). Combined biomarker assays showed higher sensitivity and specificity than cytokine or antibody assays alone. The findings from these studies suggest that using multiple biomarker types may lead to the development of improved ATB diagnostic tests.

Control of LTBI is an important milestone in the WHO End TB Strategy as this population represents a significant global reservoir of TB (54). Some *Mtb*-specific antibodies have shown promise as biomarkers of LTBI, which can be difficult to diagnose in LMICs as chest X-ray and PCR-based diagnostics are often inaccessible (99). A recent study aimed to identify antibodies that could differentiate ATB from LTBI in a cohort of *Mtb*-infected people with or without HIV co-infection (100). This large-scale analysis examined 209 *Mtb* antigens, including well-characterised antigens like LAM and antigen 85 (Ag85) and novel antigens including Rv2435.C, Rv3583 and Rv1528. This study measured *Mtb* antibody levels and IgG affinity for a selection of different Fcγ receptors (FcγRIIAR, FcγRIIB, FcγRIIIAV, and FcγRIIIB). Antigen selection was based on predictions from *in vivo* and *in vitro* models that suggested enriched transcription in the hypoxic conditions of lung granulomas (101). While *Mtb*-specific antibody levels alone could not reliably discriminate between the TB disease states, Fcγ receptor binding profiles in combination with *Mtb* antibody levels could discriminate ATB from LTBI in the study cohort, irrespective of HIV status (100). The discriminatory parameters were enriched in people with ATB and included high levels of Rv2435.C IgG1 as well as increased binding of Rv3583, Rv1528 and LAM IgG1 to FcγRIIAR, FcγRIIB and FcγRIIIAV respectively. There may be an opportunity for future development of a rapid POC diagnostic test based on some of the identified parameters (100).

The robust IgG1 response mounted against the *Mtb* growth-associated ESAT-6 and latency-associated MDP1 antigens may represent potential biomarkers in individuals with recent LTBI (102). The *Mtb* antigens ESAT-6 and CFP-10 are already used in commercially available IGRA to measure the *Mtb*-specific memory T cell response. Associations between T- and B cell responses to ESAT-6 and CFP-10 have not been studied in TB endemic LMICs and this may provide further insight into the adaptive immune response to *Mtb* infection. Antibody against the PPE17 antigen may also be a useful marker for LTBI, as anti-PPE17 IgG titres are elevated in people with LTBI compared to *Mtb* unexposed individuals (80). Modulation of the host immune response by PPE17 leads to the release of the proinflammatory cytokine tumor necrosis factor-alpha (TNF-α), which is associated with granuloma formation in TB. The PPE17 antigen may, therefore, be involved in establishing and maintaining LTBI (103, 104). Interestingly, detection of anti-PPE17 antibody was able to identify individuals with LTBI who tested negative by QuantiFERON IGRA (QFT-IGRA) with a sensitivity of 65-86% and a specificity of 100% (80). Higher PPE17-specific antibody levels were detected in people with LTBI compared to the IGRA antigens (ESAT-6 and CFP-10), suggesting that anti-PPE17 antibody may be a useful adjunct marker for LTBI.

Furthermore, high levels of IgG against the Mce1A may also help differentiate LTBI from pulmonary ATB in both adults and children with a sensitivity of 79.5% and a specificity of 84.4% (78, 105). While this Mce1A IgG test did not meet WHO sensitivity cut-offs, it performed better than other serological TB tests in children (66, 106). Antibodies against the Mce1A antigen represent a promising biomarker that could be incorporated into improved serodiagnostic tests for TB to distinguish between disease states (28, 107). Serological assays that detect multiple antibody isotypes (IgG, IgA and IgM) against different *Mtb* antigens such as LAM, HspX, and MPT32 also show increased sensitivity and specificity for ATB and LTBI, with some tests producing values of ≥ 98% for both (**Table 1**) (93–95). These POC antibody tests are successfully used for other diseases like COVID-19 and may be helpful for improving TB diagnosis in LMICs (108).

A multiplex approach may be necessary for TB serodiagnosis, as tests must account for the variability of the antibody response both within and between individuals during the course of *Mtb* infection (29). Research in broader study populations is required to determine the clinical relevance of assays for diagnosing and distinguishing ATB from LTBI. The exact definitions of TB disease states are inconsistent and vary across studies, with different diagnostic tests and patient characteristics being used to define TB groups. A more standardised diagnostic definition of TB disease states is needed to produce comparable results between studies. Furthermore, a systematic approach, based on existing immunoproteomics data, should be used to guide the selection of *Mtb* antigens and antibody isotypes measured in future work.

## 3 *Mtb* antibody therapy and prophylaxis

### 3.1 Serum and IgG transfer prophylaxis for TB

Early studies suggest that TB prophylaxis, through passive infusion of *Mtb* antibodies prior to infection, may improve clinical outcome. Serum transfer trials from the late 1800s to early 1900s showed that patients with acute and localised TB had better outcomes following serum transfer than chronic cases (109). These positive outcomes included marked reduction in clinical symptoms and bacterial load. Antibody transfer studies for TB are ongoing in animal models, with a strong focus on prophylaxis. Chen et al. (2020) infused purified human anti-AM IgG from asymptomatic individuals with LTBI or PTB patients into mice one day before and one day after *Mtb* challenge (110). The LTBI IgG led to a greater reduction in lung colony forming units (CFU) than the PTB IgG *in vivo* (110). Intratracheal administration of total human IgG before *Mtb* challenge also reduced CFU in the lungs of treated mice compared to untreated controls (111). In this study, the observed protection was mediated by *Mtb*-specific antibodies as it was lost following absorption of *Mtb*-specific gamma globulin prior to inoculation (111). Polyclonal human IgG specific for mycobacterial surface proteins was protective in a murine TB model when administered five hours before lethal *Mtb* challenge (14). Additionally, IgG isolated from humans with LTBI and highly exposed but uninfected individuals significantly reduced lung CFU compared to IgG purified from humans with ATB. These results suggest that highly *Mtb-*exposed humans may generate surface-directed *Mtb* antibodies capable of preventing ATB and possibly LTBI, and that a lack of *Mtb*-specific IgG may contribute to ATB development. Identification of the key protective antigens and antibodies will be critical to unlocking the therapeutic potential of anti-*Mtb* IgG.

Antibody-based therapies and prophylactics are important avenues for further inquiry. While complete prevention of *Mtb* infection was not observed, these studies show that antibody-based prophylactics have the potential to prevent severe TB disease. This may be especially relevant for individuals who are at high risk of developing severe TB, including people living with HIV. No prophylactic antibody studies have focused on treating TB in HIV coinfection, likely due to the complexity and limitations of modelling HIV in mice (112). Current *Mtb* prophylaxis and vaccination regimens are not optimal for individuals with HIV coinfection due to compromised immune function and drug interactions (113). Several frontline TB antibiotics (including rifamycins) are contraindicated with common antiretroviral therapies due to reduced drug efficacy, increased toxicity, and TB immune reconstitution inflammatory syndrome (114). In LMICs, where access to specific HIV/TB drug combinations is limited, alternative treatment options such as antibody-based prophylactics or therapeutics may help remove this barrier to effective management of HIV and TB coinfection. However, the utility of antibody-based therapies in settings with poor access to healthcare may be limited, as passive infusion requires trained healthcare workers and specialist equipment that may not be present in remote, resource poor areas. There are, however, large populations with access to clinics who may benefit from antibody-based prophylaxis to help limit TB spread and reduce mortality.

### 3.2 *Mtb*-specific monoclonal antibodies

A variety of monoclonal antibodies (mAbs) against major *Mtb* virulence factors, including PstS1, HBHA, HspX, LAM, MPB83 and AM, are protective in murine models of TB (15, 35, 115–120). These *Mtb*-specific mAbs are associated with a range of outcomes *in vivo* including altered granulomatous pathology of the lungs, decreased dissemination of *Mtb* to the spleen and inhibition of intracellular bacterial growth (15, 35, 115–120).

Differential glycosylation of the antibody fragment crystallizable (Fc) domain affects structure and flexibility, altering Fc receptor binding and effector functions (121, 122). Early work showed that an aglycosylated mAb could not interact with FcγRs on cells, activate complement, induce antibody-dependent cellular cytotoxicity (ADCC) or efficiently clear immune complexes (123, 124). The Fc domain of IgG contains two N-glycosylation sites at asparagine 297 (N297), one on each heavy chain. The core Fc glycan on IgG is a biantennary heptasaccaride to which fucose, sialic acid and galactose can be added (125). Afucosylated IgG has a higher affinity for FcγRIIIA and enhances ADCC activity (126–128). The addition of sialic acid to the IgG Fc domain has been shown to have anti-inflammatory effects (129). Studies have reported reduced affinity for FcγRIIIA and lower ADCC with sialylated IgG (130, 131), whereas others showed that sialylation did not influence FcγR interactions (132–134). The role of galactose on FcγR binding and downstream functions is also controversial (135–139). Chung et al. demonstrated that increased galactosylation and total sialic acid induced robust antibody-dependent phagocytosis (ADP) (140). Anti-*Mtb* IgG from individuals with LTBI showed increased galatosylation and macrophage activation compared to those with ATB (12). Comparatively, the field of IgA glycosylation is in its early stages (141). Both IgA1 and IgA2 have multiple N-glycosylation sites and IgA1 has nine potential O-glycosylation sites in the hinge region (141). The IgA N-glycan can affect thermal stability, but may not impact binding to FcαRI (142). Lower sialylation of IgA2, but not IgA1, induced pro-inflammatory functions such as neutrophil activation and cytokine production when IgA was aggregated or immobilized to form immune complexes (143). The glycosylation of *Mtb*-specific mAbs can impact functionality and different glycoforms should be investigated in pre-clinical models to identify opportunities to improve mAb efficacy.

Memory B cells isolated from humans with ATB have been used to generate protective mAbs. In one study 85 mAbs from a single patient were analysed for activity against both *Mtb* and BCG (144). Of these, two mAbs (p4-36 and p4-163) targeting the PstS1 phosphate transporter protein displayed modest anti-*Mtb* activity. The PstS1 protein is an immunodominant virulence factor in *Mtb*, and both mAbs targeted different epitopes on this antigen (145, 146). In an *ex vivo* whole blood assay, only p4-36 or p4-163 mAbs could significantly inhibit the ability of *Mtb* and BCG to grow inside human blood cells as measured by a reduction in CFU (144). In THP-1 cells, these mAbs led to opsonisation and phagocytosis of *Mtb* in an Fc-dependent manner, as an aglycosylated Fc variant of the IgG1 mAbs did not exhibit the same activity as wild type IgG1. Furthermore, blocking of macrophage FcγRs (FcγRIIIA, FcγRIIA, and FcγRIIB in combination) abrogated the observed *Mtb* growth inhibition by p4-36 and p4-163. Depletion of T cells and MHC class II had no effect on mAb activity, suggesting that these mAbs act in a T cell independent manner unlike some previously described anti-*Mtb* mAbs (14, 144). Enhanced uptake of *Mtb* by macrophages (via mAb-FcγR interactions) was observed in these cell-based assays, but the mAbs did not increase bacterial load and appeared to restrict *Mtb* growth inside the macrophage through an undefined mechanism (144). Prophylactic administration of the p4-36 or p4-163 mAbs prior to aerosol infection with *Mtb* resulted in a 0.5 log reduction in lung bacterial CFU compared to untreated mice. The discovery of protective antibodies against an immunodominant virulence factor like PstS1 represents a significant step forward and could inform development of therapeutics as well as novel vaccines. Further characterisation of the mechanisms that underpin the *in vivo* efficacy of anti-PstS1 mAbs may provide key evidence for a protective role of antibodies during *Mtb* infection.

Two HspX-specific IgA mAbs (TBA61 and 2E9) were tested for prophylactic activity in mice, via either intranasal or intratracheal delivery (115, 147). The 2E9 human IgA1 mAb was given with IFN-γ, to FcαRI transgenic mice and the TBA61 murine IgA mAb was administered to non-transgenic mice (115). Both mAbs significantly reduced lung CFU and pathology in mice challenged with a lethal or sublethal dose of *Mtb* (115, 118, 120). Protection by TBA61 was at least partially isotype-specific, as an IgG1 mAb with the same specificity was less effective at reducing lung CFU (147). Since mice do not have a known homolog of human FcαRI, the observed TBA61-mediated protection was Fc-independent (42, 148). The HspX-specific IgA mAbs were not capable of clearing *Mtb* in the non-transgenic mouse model, but mice differ from humans in both Fc receptor expression and cellular distribution. Potential functions of the HspX-specific mAbs include fragment antigen-binding (Fab)-mediated neutralization, which may prevent critical HspX functions during *Mtb* infection such as intracellular survival and resilience to stressors (149–151). Murine models may not accurately reflect the Fc-mediated effector functions of human mAbs, limiting the translation of these results into therapeutic applications (30, 152). In addition to lacking a homolog of human FcαRI, the murine ortholog of human FcγRIIIa, FcγRIV, is not expressed on NK cells (42, 115, 148). These differences have important implications when using mice to model human IgA and IgG Fc-mediated effector functions like NK cell activation and antibody dependent cellular cytotoxicity (ADCC) (153). The use of Fc receptor transgenic or humanised mice for passive infusion studies represents a more suitable model system for future *Mtb*-specific mAb studies (115, 154). Further, most studies use the laboratory type-strain of *Mtb* (H37Rv) for *in vivo* challenges (17), but H37Rv does not elicit the same immune response and pathology as clinical *Mtb* isolates (155, 156). Improved animal models of TB that better represent human infection and humoral responses are necessary to advance the translation of mAb therapies.

A combined immunotherapy (CIT) strategy with IFN-γ or anti-IL-4 further enhanced TBA61 and 2E9 IgA protection in mice (157, 158). Use of CIT may also prevent post-chemotherapy *Mtb* infection relapse, and 2E9 IFN-γ CIT is effective against multidrug resistant-TB (MDR-TB) (115, 157, 159). These outcomes suggest that some mAbs could form part of an adjunct treatment regimen for MDR-TB, which is becoming increasingly challenging to treat. Effective and timely treatment of MDR-TB is vital for limiting spread in vulnerable communities. Prophylactic use of *Mtb*-specific mAbs alongside antibiotic treatment may help to discourage the spread of MDR-TB-causing bacteria by eliminating the resistant *Mtb* bacilli before they can cause post-treatment relapse (159, 160). While promising in animal models, these studies show *Mtb*-specific mAbs are clearly most effective when administered before or early after *Mtb* infection (15, 115, 147, 158). A study analysing temporal factors that affect mAb treatment found that prophylactic administration of the TBA61 mAb was required for protection in mice (158). Therefore, *Mtb*-specific mAbs may not have broad clinical applications as diagnosis of *Mtb* is difficult and slow in LMICs where MDR-TB is most widespread (161). Access to prophylactic mAbs is also limited in these areas due to their high cost, and the need for healthcare infrastructure to deliver infusions. Targeted administration to high-risk groups such as immunocompromised and treatment resistant individuals may provide the most benefit. Regimens including *Mtb*-specific IgA mAbs and CIT may be useful for improving TB outcomes in humans but require further examination in transgenic mice and NHPs that better model human immunity.

Anti-LAM and anti-AM mAbs have also been tested in murine models of TB (13, 117, 119). Pre-incubation of *Mtb* with an AM-specific IgG3 mAb (9d8) prolonged survival in healthy and immunocompromised mice infected intratracheally with a lethal dose of *Mtb* (119). Mice infected with *Mtb* that was pre-incubated with the 9d8 mAb had enhanced granuloma formation, which constitutes a protective effect, but there was no observed reduction in CFU in the murine lung, spleen, or liver. Prophylactic administration of 9d8 at 48 hrs, 24 hrs, and 4 hrs prior to *Mtb* challenge also did not impact survival (119). However, granuloma formation and *Mtb* burden outside of the lungs were not examined in the mice infused directly with 9d8 mAb, so evidence of prophylactic effects may have been missed. A LAM-specific IgG1 mAb (SMITB14) has also shown some efficacy in a murine TB model (117). In this study, the mice were either intravenously inoculated with SMITB14 mAb one hour prior to *Mtb* infection or given a SMITB14 mAb-*Mtb* mixture. The mice infused with SMITB14 mAb before *Mtb* infection had a significant reduction in bacterial CFUs in the lungs, livers and spleens and had lower CFUs compared to mice that received the SMITB14-*Mtb* mixture (117). The Fab domain of the SMITB14 mAb administered alone was prophylactic, suggesting that neutralization may be a primary mechanism of SMITB14 protection. An *in vitro* study also found human anti-LAM IgA mAbs decreased *Mtb* infection of human lung epithelial (HLE) and monocytic THP-1 cell lines (13). The observed reduction in *Mtb* infection was likely Fc-independent as IgA FcαR is not expressed on HLE cells and the use of FcαR-expressing THP-1 cells did not further decrease *Mtb* infection (13).

The testing of *Mtb*-specific mAbs in murine models, particularly in CIT, indicates that this modality may be a valuable tool to investigate mechanisms of humoral immunity in human TB. These *Mtb*-specific mAbs have the potential to treat patients with compromised cellular immunity, shorten treatment regimens, reduce relapse after chemotherapy and provide an alternative for drug-resistant TB cases (115, 162). The use of improved animal models of TB immunity and more clinically relevant *Mtb* strains for *in vivo* testing will help advance *Mtb*-specific mAbs as future prophylactic and therapeutic agents.

## 4 TB antibodies and vaccines

### 4.1 Antibody response to Bacille Calmette-Guérin

To date, BCG is the only approved vaccine for TB, but it has demonstrated limited protection against PTB and reactivation of LTBI in adults (163). Vaccination with BCG is able to prevent severe TB disease and *Mtb* dissemination in children, and in TB endemic areas BCG can halve mortality in the first 6-12 months of life (164). However, the lack of a protective vaccine against *Mtb* in the wider human population represents a significant hurdle to the control of TB globally (30). The BCG vaccine and the majority of candidate TB vaccines focus on enhancing cell-mediated immunity (CMI) against *Mtb*. Although novel TB vaccines are in development, some groups have instead focused on enhancing the protection provided by BCG. Various strategies to improve the efficacy of BCG have been investigated such as the addition of boosters that complement BCG, altering the route of BCG delivery and the creation of recombinant BCG vaccines (165).

In addition to increasing *Mtb*-specific CMI, the BCG vaccine also induces mycobacterium (*Mb*)-specific antibody responses similar to those elicited by *Mtb* infection (18). Standard intradermal BCG vaccination generates a strong *Mb*-specific IgG and IgM response in humans, including anti-LAM and anti-AM antibodies (18, 166–169). The subclass distribution of *Mtb*-specific IgG produced following BCG vaccination is skewed towards IgG1-3 (169). The IgG induced by BCG vaccination has been shown to increase phagocytic uptake, enhance phagolysosomal fusion, inhibit *Mtb* growth and increase *Mb*-specific CD4+ and CD8+ T cell proliferation and IFN-γ production *in vitro* (18, 168). Alternative routes of BCG administration have also been explored. In humans, mucosal administration of BCG significantly increased anti-LAM IgA in serum and nasal washes (18, 167). Mucosal BCG vaccination in rhesus macaques also boosted anti-purified protein derivative (anti-PPD) IgA in bronchoalveolar lavage (BAL) and serum, but the reduction in lung CFU and pathology after *Mtb* challenge did not correlate with IgA levels (170).

The antibodies elicited by BCG vaccination may be associated with TB protection in children but the lack of efficacy in adults suggests that BCG-induced antibodies do not provide sustained protection (36, 171, 172). Some trials aim to enhance BCG-mediated protection by adding novel subunit vaccines as boosters (like H4:IC31 and H56:IC31), although some of these subunit formulations are also being trialled as standalone vaccines (173–176). The H4:IC31 subunit vaccine consists of a fusion protein of two mycobacterial antigens Ag85B and TB10.4 (H4), while H56:IC31 is composed of a fusion protein of Ag85B, ESAT-6, and Rv2660c (H56). These novel subunit vaccines induced anti-H4 or -H56 antibody responses in humans, dominated by the IgG1 and IgG3 subclasses (173, 177). In NHP models, the inclusion of a H56:IC31 booster (post-vaccination with BCG) improved containment of *Mtb* infection, reduced lung pathology, and decreased *Mtb* dissemination from the lungs (176).

The efficacy of these TB booster vaccines in humans is not yet known, but they have been tested for safety and immunogenicity in phase I clinical trials (173, 175, 177). Future trials of TB subunit vaccines should include detailed serological analyses of *Mtb*-specific antibodies produced and their functions, as this may provide insight into the mechanisms of protection that underpin AMI to TB. Further trials in animal models may also be necessary to thoroughly assess the specific role of anti-H4 and -H56 antibodies on lung pathology, survival, and bacterial load. Administration of BCG with booster vaccines has the potential to provide greater and more sustained protection than BCG alone, and this may be attributed to the induction of both AMI and CMI.

### 4.2 Anti-*Mtb* antibody response to novel vaccines

There are a large number of TB vaccines in pre-clinical development and early clinical trials (178). The majority of TB vaccine studies focus on CMI, and none of the candidate TB vaccines that have progressed to clinical trials are specifically designed to elicit *Mtb* antibodies (53). However, numerous studies have shown an association between vaccine-induced *Mtb* antibodies and protection from severe TB (53). These findings suggest that some candidate TB vaccines may stimulate AMI capable of reducing TB disease severity in cooperation with cellular immunity. Most novel TB vaccines appear to induce detectable *Mtb*-specific antibodies (179). The antibody response to new TB vaccines is often reported in clinical trials, but analysis of *Mtb* antibodies is often limited to correlations with T helper cell type (Th1 vs Th2) and cytokine responses (180, 181). Few TB vaccine studies investigate the protective or pathological roles of *Mtb* antibodies *in vitro* or in animal models (16).

A phase IIb clinical trial for the viral vectored modified Vaccinia Ankara virus 85A (MVA85A) vaccine in BCG-vaccinated infants (aged 4-6 months) induced anti-*Mtb* antibodies that were associated with TB protection (182). While the original trial did not measure the antibody response, a retrospective case-controlled review found that increased Ag85A-specific IgG was associated with a decreased risk of TB disease (183). Interestingly, the MVA85A vaccine was designed to elicit a strong CD4+ T cell response but failed to be more efficacious than the current BCG vaccine. As such, there is a growing interest in developing TB vaccines that stimulate the humoral and cellular arms of adaptive immunity. A novel adenoviral vectored vaccine ChAdOx1 85A enhanced MVA85A-mediated protection and induced Ag85A-specific IgG in BCG-vaccinated adults (184, 185). The *Mtb* Ag85 complex is also the target of several DNA vaccines, which induced high levels of IgG1 and IgG2 against Ag85A or Ag85B (186–189). Recent advances in the development of viral-vectored vaccines for COVID-19 could be applicable to TB, alone or as a booster for BCG (190). A MVA vector expressing the HspX antigen was tested as a booster for BCG, and significantly reduced bacillary load in guinea pig models (191). Clinical trials for several viral vectored TB vaccines are ongoing, and the results from the MVA85A trial should encourage more detailed serological analyses to investigate whether humoral immunity is influencing vaccine efficacy (190).

The anti-LAM and -AM IgG induced by three separate TB subunit vaccines may confer some protection against *Mtb* infection (16, 36, 192, 193). One vaccine consisted of AM conjugated to Ag85b (AM-Ag85b) or *B. anthracis* protective antigen (AM-PA) (16). Mice immunised with AM-Ag85b had longer survival time, reduced lung pathology, and reduced CFU in the lung and spleen following lethal *Mtb* challenge compared to BCG (16). Survival time was increased by anti-AM antibodies as mice vaccinated with AM-Ag85b had longer survival times than mice that received Ag85b alone. However, both anti-AM and anti-Ag85b antibodies were required to achieve the full protective effect since AM-Ag85b vaccinated mice showed a greater reduction in lung CFU compared to AM-PA or Ag85b vaccinated groups. The protective effects of anti-Ag85 antibodies in AM-Ag85b vaccinated mice supports the observed association between high anti-Ag85A antibodies and a decreased risk of TB disease in MVA85A vaccinated children (16, 183). Induction of antibodies targeting the *Mtb* Ag85 complex appears to be a rational avenue for the development of protective TB vaccines. Future trials for the MVA85A vaccine should include assays to quantify anti-Ag85 antibodies as they represent a potential immune correlate of protection.

The live attenuated *Mtb* vaccine candidate *MTB*VAC is designed for newborns (194). This vaccine is composed of an *Mtb* isolate (Mt103) attenuated by the deletion of the *phoP* and *fadD26* genes that encode important virulence factors (195). Mucosal vaccination with *MTB*VAC in rhesus macaques induced PPD-specific IgA, IgG, and IgM antibodies (196). The BAL IgG and IgM antibodies produced by mucosal *MTB*VAC vaccination showed increased binding to *Mtb* compared to BAL antibodies from subcutaneous vaccination (196). The BAL antibodies from mucosal vaccination with *MTB*VAC also increased ADP of *Mtb* by THP-1 cells *in vitro* (196). The specificity of these antibodies is currently unknown and should be elucidated to allow more focused analysis of their role, if any, in *MTB*VAC protection. Additional testing of *MTB*VAC-induced antibodies for Fc-mediated effector functions, such as NK cell activation, may further advance our understanding of their potential mechanisms of protection.

## 5 Conclusions

The WHO End TB Strategy outlines key pillars that are required to end TB by 2030, and one of these pillars is the development of new tools to diagnose, treat and manage TB (2, 54). The antibody response to *Mtb* infection and vaccination represents a potential avenue for developing these TB management strategies. While the functions of TB antibodies are not fully understood, it is clear that *Mtb*-specific antibodies have a role in the course of *Mtb* infection and disease. There is, however, a lack of available data to establish protective associations between the *Mtb* antibody profile and TB disease outcomes. Antibodies against *Mtb* may have useful applications in the diagnosis, treatment, and control of TB. Serology-based multiplex assays for TB have shown high sensitivity and specificity and could distinguish between different TB disease presentations. Identification of optimal *Mtb* antibody specificities and isotypes for serodiagnosis of TB is ongoing. Studies in animal models suggest that antibody-based prophylaxis may be useful for preventing severe TB in at-risk populations such as people living with HIV. New candidate TB vaccines can induce anti-*Mtb* antibodies that may work in conjunction with CMI to improve protection from *Mtb* infection in broader populations. Antibodies show some promise for the management and prevention of TB, but a greater understanding of TB antibodies is needed to progress antibody-based diagnostics and therapeutics beyond the research arena and into the clinic.

## Author contributions

SM: Conceptualization, Writing – original draft. CR: Conceptualization, Supervision, Writing – review & editing. JW: Conceptualization, Writing – review & editing. HV: Supervision, Conceptualization, Funding acquisition, Writing – review & editing.
